# Indirect Virus Transmission in Cluster of COVID-19 Cases, Wenzhou, China, 2020

**DOI:** 10.3201/eid2606.200412

**Published:** 2020-06

**Authors:** Jing Cai, Wenjie Sun, Jianping Huang, Michelle Gamber, Jing Wu, Guiqing He

**Affiliations:** Wenzhou Sixth People’s Hospital, Wenzhou Central Hospital Medical Group, Wenzhou, China (J. Cai, J. Huang, G. He);; The Second Affiliated Hospital of Fujian Traditional Chinese Medical University, Fuzhou, China (W. Sun);; Robert Stempel College of Public Health and Social Work, Florida International University, Miami, Florida, USA (W. Sun);; Shenandoah University, Winchester, Virginia, USA (M. Gamber);; Huashan Hospital, Fudan University, Shanghai, China (J. Wu)

**Keywords:** coronavirus, viruses, COVID-19, severe acute respiratory syndrome coronavirus 2, SARS-CoV-2, cluster, aerosolization, Wenzhou, China, 2019 novel coronavirus disease, respiratory infections, zoonoses

## Abstract

To determine possible modes of virus transmission, we investigated a cluster of coronavirus disease cases associated with a shopping mall in Wenzhou, China. Data indicated that indirect transmission of the causative virus occurred, perhaps resulting from virus contamination of common objects, virus aerosolization in a confined space, or spread from asymptomatic infected persons.

Severe acute respiratory syndrome coronavirus 2 (SARS-CoV-2), the causative agent of coronavirus disease (COVID-19), is presumed to spread primarily via respiratory droplets and close contact. However, these transmission modes do not explain all cases. To determine how the virus may have spread among a cluster of COVID-19 cases associated with a shopping mall in Wenzhou (a city with 8 million residents), China, we monitored and traced close contacts and hypothesized possible transmission modes. We analyzed clinical and laboratory data for cases by using real-time reverse transcription PCR (*1*). The study was approved with written consent from the Ethics Committee of Wenzhou Central Hospital and written informed consent from all case-patients.

On January 20, 2020, a 23-year-old man (patient E) sought care at a hospital after 11 days of fever and headache. On January 21, COVID-19 was confirmed for patient E and his co-worker, patient G. The Wenzhou Center for Disease Control and Prevention traced and tested their contacts, and by January 28, COVID-19 was confirmed for 7 persons (patients A–G) from the same office (on floor 7).

Patient A, a 30-year-old woman, the only case-patient who indicated that she had been in Wuhan, China, returned from Wuhan on December 18, 2019. On January 15–16, 2020, she had a fever, but symptoms resolved without treatment. Despite symptom resolution, on January 30 she was confirmed to have SARS-CoV-2 infection. If patient A is the index patient, infected in Wuhan, her incubation period would have been 28 days, which would be extremely long, according to updated information (W.J. Guan et al., unpub. data, https://www.medrxiv.org/content/10.1101/2020.02.06.20020974v1). Asymptomatic carrier transmission has been reported for SARS-CoV-2 (*2*); hence, patient A could have been screened as a close contact during her incubation period and then hospitalized on the basis of a positive test (PCR) result only. However, her clinical symptoms did not appear until after hospitalization. Because persons with asymptomatic COVID-19 can spread the virus, patient A also could have been an asymptomatic carrier with a persistent infection (*3*).

On January 22, the mall was shut down. During January 19–February 9, COVID-19 was diagnosed for 7 mall staff from floors B1–3 and for 10 mall customers. Close contacts associated with the mall were traced, and COVID-19 was confirmed for 11 persons. Sixteen patients had had direct contact with other patients or had gone shopping in the mall. The average incubation period was 7.3 (range 1–17) days.

The mall has 8 floors above ground and several basement levels; floors B1 to 6 are commercial shopping space, and floor 7 contains shopping and office space. We created an illustration showing the floors where the eventual COVID-19 case-patients worked or shopped, along with dates of symptom onset, potential incubation periods, symptom durations, confirmed times of positive diagnosis, and times of discharge (Figure, panel A).

**Figure:**
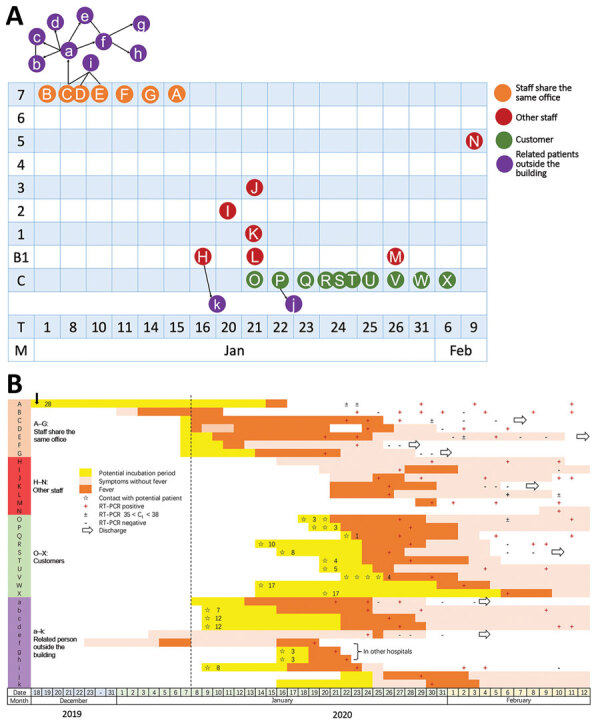
Cluster of COVID-19 cases associated with a shopping mall in Wenzhou, China. A) Distribution of COVID-19 case-patients by mall floor, time, and internal relationship. B) Dates of symptom onset, confirmed test results, and hospitalization information. Numbers within yellow bars indicate length of incubation period. Black vertical arrow indicates date when patient A returned from Wuhan, China. B1–7, mall floors; C, customer; COVID-19, coronavirus disease; C_t_, cycle threshold; T, date of symptom onset; M, month; RT-PCR, reverse transcription PCR.

Except for those who had been on floor 7, all other case-patients denied direct close contact with other case-patients. The possibility of customers being infected from other sources cannot be excluded. However, most customers reported early symptom onset in a concentrated time frame (Figure, panel B). We found no convincing evidence of definitive transmission pathways in this building. Patients A–G (Figure, panel A) worked in the same room on floor 7. Other case-patients who had been on other floors denied any direct contact with confirmed patients from floor 7, but they shared common building facilities (e.g., restrooms, elevators). Also, staff from floor 7 visited shops on other floors daily.

Until now, no evidence has shown that SARS-CoV-2 can survive outside the body for long. However, Middle East respiratory syndrome coronavirus demonstrates high robustness and a strong capability to survive outside the body and can remain infectious for up to 60 minutes after aerosolization (*4*). Hence, the rapid spread of SARS-CoV-2 in our study could have resulted from spread via fomites (e.g., elevator buttons or restroom taps) or virus aerosolization in a confined public space (e.g., restrooms or elevators). All case-patients other than those on floor 7 were female, including a restroom cleaner, so common restroom use could have been the infection source. For case-patients who were customers in the shopping mall but did not report using the restroom, the source of infection could have been the elevators. The Guangzhou Center for Disease Control and Prevention detected the nucleic acid of SARS-CoV-2 on a doorknob at a patient’s house (*5*), but Wenzhou Center for Disease Control and Prevention test results for an environmental sample from the surface of a mall elevator wall and button were negative. 

We cannot exclude the possibility of unknown infected persons (e.g., asymptomatic carriers) spreading the virus. However, according to screening protocols implemented by the Wenzhou Center for Disease Control and Prevention, we traced all close contacts and included all patients with positive PCR results, including the asymptomatic carrier (patient A), in this study. Our findings appear to indicate that low intensity transmission occurred without prolonged close contact in this mall; that is, the virus spread by indirect transmission.
